# Laser particles with omnidirectional emission for cell tracking

**DOI:** 10.1038/s41377-021-00466-0

**Published:** 2021-01-25

**Authors:** Shui-Jing Tang, Paul H. Dannenberg, Andreas C. Liapis, Nicola Martino, Yue Zhuo, Yun-Feng Xiao, Seok-Hyun Yun

**Affiliations:** 1 Harvard Medical School and Wellman Center for Photomedicine, Massachusetts General Hospital, Boston, MA USA; 2 State Key Laboratory for Mesoscopic Physics and Frontiers Science Center for Nano-optoelectronics, School of Physics, Peking University, 100871 Beijing, China; 3 Harvard-MIT Health Sciences and Technology, Massachusetts Institute of Technology, Cambridge, MA USA

**Keywords:** Optical materials and structures, Lasers, LEDs and light sources

## Abstract

The ability to track individual cells in space over time is crucial to analyzing heterogeneous cell populations. Recently, microlaser particles have emerged as unique optical probes for massively multiplexed single-cell tagging. However, the microlaser far-field emission is inherently direction-dependent, which causes strong intensity fluctuations when the orientation of the particle varies randomly inside cells. Here, we demonstrate a general solution based on the incorporation of nanoscale light scatterers into microlasers. Two schemes are developed by introducing either boundary defects or a scattering layer into microdisk lasers. The resulting laser output is omnidirectional, with the minimum-to-maximum ratio of the angle-dependent intensity improving from 0.007 (−24 dB) to > 0.23 (−6 dB). After transfer into live cells in vitro, the omnidirectional laser particles within moving cells could be tracked continuously with high signal-to-noise ratios for 2 h, while conventional microlasers exhibited frequent signal loss causing tracking failure.

## Introduction

Laser particles (LPs)—micro- and nanolasers in the form of particles dispersible in aqueous solution—have emerged as a new promising optical tool in the life sciences^1–8^. In comparison to conventional photoluminescent probes, such as fluorescent molecules, dye-doped microbeads, and gold nanoparticles, the laser emission from LPs has a few distinctive characteristics. The most striking feature is its narrow spectral bandwidth of <0.3 nm. This feature makes LPs an attractive choice for spectrally multiplexed tagging of cells so that individual cells in a heterogeneous population can be tracked in in vitro experiments, in a live animal, or across different single-cell analysis instruments, for example, from microscopy to single-cell sequencing^6,7,9^. Recently, intracellular microdisk LPs were used to track 5000 cells in a tumor spheroid, a number that can be scaled to more than millions of cells by combining multiple microdisks each with a distinctive spectral peak^7,9^.

Another intrinsic feature of LPs is that their output emission occurs in specific directions determined by the lasing cavity mode. Microdisk LPs supporting whispering gallery modes (WGMs) emit predominantly in the plane of the cavity resonance^10–13^. The directionality of laser emission, however, hinders the reliable optical reading of LPs when their orientations with respect to optical instruments change. This is a general problem in almost all applications of LPs, as this new type of laser is intended to operate with arbitrary, often freely moving, orientations. Cell tracking represents this situation. The orientations of LPs in a cell are arbitrary and tend to vary over time as the cell moves. During tracking, this can cause random intensity fluctuations and frequent loss of the measured laser signal, making it difficult to detect and identify LPs reliably over time. We encountered this problem in our previous study of spheroids in vitro. The light scattering in biological tissues does not mitigate this problem because high-resolution spectral readout of LP emission requires confocal detection of essentially non-scattered, or minimally scattered, light. Furthermore, the detection of LPs in instruments with dynamic environments, such as microfluidic channels^14^, would severely suffer from the angular dependence of the emission. Therefore, addressing the directionality of the laser emission would have a high impact on the broad utility of LPs.

Previous works on microlasers for on-chip applications mainly focused on directing the in-plane emission of WGM microlasers in a specific direction by introducing boundary deformations^11,15–19^, diffraction gratings^20–22^, or scatterers^23–26^. Nevertheless, omnidirectional laser emission into 4*π* steradians has neither been attempted nor demonstrated. Liquid-crystal microspheres^27,28^ have the potential for omnidirectional lasing, but the large resonator size (tens of micrometers) required to form a radial grating is unsuitable for intracellular applications.

Here, we demonstrate omnidirectional emission from microdisk LPs by incorporating light scattering into the cavity. Among the various approaches we have explored, two designs of omnidirectional LPs (OLPs) are described here: one introduces boundary defects into the cavity design, and the other uses nanoparticles attached around the resonators. The laser power collected from our OLPs varies by <10 dB as a function of their orientation, while this same variation exceeds 24 dB for conventional microdisk LPs (CLPs). We find that despite the strong scattering and large aspect ratio of both OLP designs, single-mode lasing is realized with a low lasing threshold and a narrow linewidth nearly independent of their orientation. We have applied one of the designs to produce OLPs in large quantities for a proof-of-concept demonstration of reliable single-cell tagging and blinking-free cell tracking. The traces of OLPs based on their output spectra in live cells show a high signal-to-noise ratio (SNR) in every single frame for 2 h in practical settings, while with CLPs, the signal is below the noise level in many frames.

## Results

### **I**n-plane emission of microdisk LPs

**Fig. 1:**
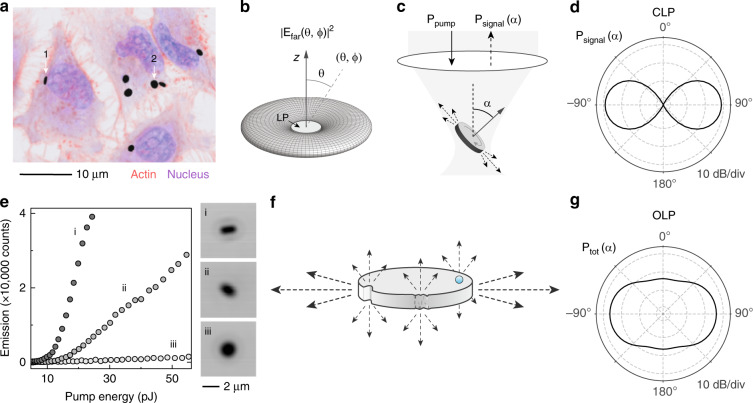
Orientation-dependent laser emission of microdisk LPs. **a** Cells tagged with LPs. Two representative disk orientations are labeled. **b** Far-field radiation pattern $\left| {E_{{\mathrm{far}}}(\theta ,\phi )} \right|^2$ of the 10th-order TE WGM of a conventional microdisk laser. **c** Schematic of the pumping and collection geometry. **d** Simulated *P*_signal_(*α*) of a CLP. **e** *P*_signal_ versus pump energy *P*_pump_ for three CLPs suspended in a hydrogel with different orientations. Insets: Corresponding optical images (i, ii, iii). **f** Illustration of various strategies for achieving omnidirectional emission: A deformation (notch) on the boundary of the microdisk, surface roughness, or high-index nanoparticles attached to the microdisk can redirect a portion of the lasing emission into the normal direction via elastic scattering. **g** Simulated *P*_tot_(*α*) of an OLP with a single 200-nm-size notch scatterer

We produced InGaAsP semiconductor microdisk lasers as described previously^7^. The microdisks were released from the substrate by wet chemical etching and loaded into cells. The orientation of LPs in tagged cells is arbitrary (Fig. 1a) and tends to vary rapidly over time as the cells move (Supplementary video).

Consider a typical semiconductor microdisk laser with a diameter of 2 μm and a thickness of 200 nm. Finite-element-method (FEM) simulations identify the transverse-electric (TE, meaning *E*_*z*_ = 0) WGM at a representative wavelength of 1270 nm (Fig. S1a) as the mode with the highest passive quality factor (radiation-limited *Q*_rad_ > 10^4^) within the gain bandwidth of the semiconductor material. Therefore, it is the fundamental TE mode that lases. The transverse-magnetic (TM) mode has a lower quality factor and does not reach the lasing threshold easily because of gain competition with the TE mode. As intracavity light circulates along the periphery of the resonator, a small portion leaks out of the side of the disk with each reflection, resulting in emission predominantly in the plane of the disk (Fig. 1b). The far-field E-field pattern of the cavity mode can be calculated as^12^ (Supplementary Note 1) $E_{{\mathrm{far}}}(\theta ,\phi ) \approx \left| {{\mathrm{sin}}\theta } \right|^9e^{im\phi }$, where *m* (=10) is the mode order, and $(\theta ,\phi )$ are defined in the spherical coordinate system fixed to the reference frame of the LP (Fig. 1b).

The output emission is collected with a finite numerical aperture (NA). For a microdisk LP tilted with respect to the viewing axis by an angle *α*, the power collected is given by

1 $$P_{{\mathrm{signal}}}\left(\upalpha,{\mathrm{NA}} \right) \propto \int_{\mathrm{{\Omega} }} \left| {E_{{\mathrm{far}}}\left( {\theta ,\phi } \right)} \right|^2{\mathrm{sin}}\theta {\mathrm{d}}\theta {\mathrm{d}}\phi$$

where the integration is performed over the solid angle Ω defined by a cone with half angle asin(NA/n) centered on *α* (Fig. 1c), and n is the refractive index of the surrounding material (*n* = 1.33 for an aqueous medium). When standing waves are formed by two counterpropagating WGMs (Fig. S1g), the lobed azimuthal structure in the far-field intensity profile^29^ will be approximately averaged out provided that the collection NA is >0.5*πn*/*m*, or >0.2 for *m* = 10.

Figure 1d illustrates that the simulated power *P*_signal_ collected by an objective with an NA of 0.45 is highly dependent on the tilt angle *α* of the disk. The acceptance angle of light collection in the aqueous medium is asin (NA/*n*) = 19.8° with Ω = 0.37 sr. Approximately, *P*_signal_∝sin^10^(*α*) for NA = 0.45. The maximum intensity is obtained at *α* = 90°, corresponding to a microdisk LP oriented along the measurement direction (e.g., disk 1 in Fig. 1a), while the minimum intensity is obtained at *α* = 0° from a flat disk (e.g., disk 2 in Fig. 1a). To quantify this angle dependence, we calculated the ratio *R* of the minimum and maximum powers, or the dynamic range of intensity, from the results of Eq. (1). The ratio measured in the FEM simulation is −37 dB for NA = 0.45.

We may define the criterion for omnidirectionality as *R* > 0.01 or −20 dB since this could result in an adequate SNR when a spectrometer with a typical dynamic range of 30 dB is used. We consider *R* > 0.1 to be highly omnidirectional. It should be noted that *R* is a function of the NA for light collection (Supplementary Note 2, Fig. S10). *R* > 0.01 and *R* > 0.1 could be achieved in principle using NA > 1.1 and NA > 1.23, respectively. However, such a high NA may not be applicable under certain experimental conditions and is not possible without immersion lenses. In the following, we characterize the angle-dependent emission for NA = 0.45, which gives a diffraction-limited volume encompassing an entire microdisk.

To experimentally characterize the angle dependence, we suspended microdisk LPs in a curable gelatine hydrogel (Matrigel, *n* = 1.334) with fixed, random orientations and examined them under a laser-scanning confocal microscope combined with a pump laser (1064 nm wavelength, 3 ns pulse width, 2 MHz repetition, and 2.9 μm focal beam size) and a high-resolution spectrometer (Materials and methods). Upon optical pumping above their lasing threshold, the microdisk LPs exhibited single-mode emission with a sub-nm linewidth (Fig. S2). In Fig. 1e, the input-output curves of three representative LPs illustrate a distinct dependence on the orientation of the disks. Significantly more lasing light was collected from vertically oriented disks (Fig. 1e-i) than from flat disks (Fig. 1e-iii), as evidenced by the increased slope or “slope efficiency” of the input-output curve above the threshold (~10 pJ).

### Omnidirectional-emitting laser particles (OLPs)

Although the output emission of a CLP is directional because of the innate geometry of its cavity structure, it is possible to transform the emission pattern by introducing perturbations. For example, surface roughness, boundary deformations or nanoscale scatterers could redirect part of the lasing light to directions out of the disk plane via elastic scattering (Fig. 1f). As the electric field of the TE mode lies primarily in the plane of the disk (Fig. S3a), the Rayleigh-scattered pattern *P*_sc_(*α*) ∝ 1 + cos^2^*α* has a maximum in the direction perpendicular to the disk plane (Supplementary Note 3). Combining the predominantly in-plane emission from the microdisk *P*_0_(*α*) and the predominantly out-of-plane scattering from the perturbations *P*_sc_(*α*), the total pattern can be expressed as

2 $$\begin{array}{*{20}{c}} {P_{{\mathrm{tot}}}\left( \alpha \right) = \left( {1 - s} \right) \cdot P_0\left( \alpha \right) + s \cdot P_{{\mathrm{sca}}}\left( \alpha \right)} \end{array}$$

$$\propto \left( {1 - s} \right) \cdot c_0{\mathrm{sin}}^{10}\alpha + s \cdot c_1\left( {1 + {\mathrm{cos}}^2\alpha } \right)$$

where *s* represents the fraction of light scattered, *c*_0 _≈ 0.75 and *c*_1_ ≈ 0.21 (Supplementary Note 3). Therefore, the min-max ratio becomes *R* ≈ 0.56s/(1 − 0.72s). The criteria of *R* > 0.01 and *R* > 0.1 are satisfied when *s* > 0.018 and *s* > 0.16, respectively. Figure 1g shows an FEM simulation result for an LP with a single defect: a 200-nm semicircular notch. The calculated *P*_tot_(*α*) is highly omnidirectional with *R* ≈ 0.1 or −10 dB.

**Fig. 2:**
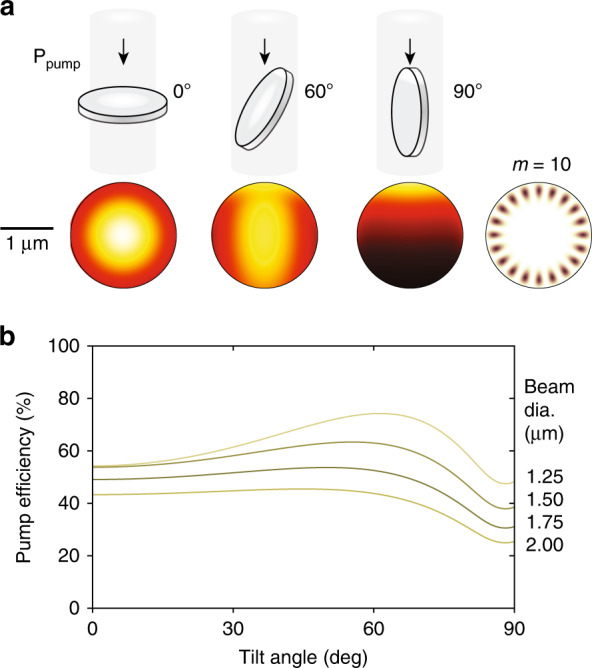
Orientation dependence of the pump efficiency. **a** Spatial distribution of absorbed pump energy at different tilt angles, and intensity profile of the cavity mode (*m* = 10). The pump beam has a Gaussian profile with a size of 1.5 μm. **b** Pump efficiency versus tilt angle for pump beam diameters of 1.25, 1.5, 1.75, and 2 μm

Thus far, we have considered the light collected from a microdisk. As the LP changes its orientation with respect to the excitation and collection optics (Fig. 1c), not only its emission but also the absorption of pump light in the LP will change. This can affect the threshold energy and, thereby, the total magnitude of the output emission as a function of the tilt angle, contributing to the angle dependence of the collected light. To consider this effect, we calculated the amount of pump absorption. We used a geometrical model of light propagation in the microdisk with Fresnel reflection at the semiconductor-water interfaces and absorption in the material following a Lambert-Beer profile with an absorption coefficient of 1.75 × 10^4^ cm^−1^. The pump beam was modeled with a Gaussian profile centered on its axis tilted at an angle *α*. Figure 2a shows the spatial distribution of the pump absorption at different disk orientations for a pump beam diameter of 1.5 μm at full width at half maximum (FWHM), which corresponds to our experimental conditions. The pump efficiency *η*_*p*_ is defined as the overlap between the absorbed pump energy distribution *ρ*(*α*) and the mode profile $\left| u \right|^2$ of the cavity resonance normalized by the same overlap integral in the case of a uniform pump distribution (*ρ*_*U*_), $\eta _{\mathrm{P}}\left( \theta \right) = \int_{{\mathrm{cavity}}} \rho (\theta )\left| u \right|^2{\mathrm{d}}V/\int_{{\mathrm{cavity}}} \rho _U\left| u \right|^2{\mathrm{d}}V.$ The angle dependence of pump efficiency *η*_*p*_ is shown in Fig. 2b. The lasing threshold, in the first approximation, is proportional to 1/*η*_*p*_. For a beam size of 1.5 μm, the angular dependence of the threshold energy is only 20%.

### LPs with surface roughness

Nanometer-scale imperfections behave as Rayleigh scatterers and couple the resonant optical modes into far-field radiation^30,31^. Conventional wisdom states that one should strive to reduce such imperfections during fabrication to maximize the quality factor of the laser cavity and thereby reduce the lasing threshold. We hypothesized that the sidewall roughness arising from reactive-ion etching (RIE) can be intentionally introduced to reduce the emission directionality of microdisk LPs without significantly affecting the lasing threshold.

**Fig. 3:**
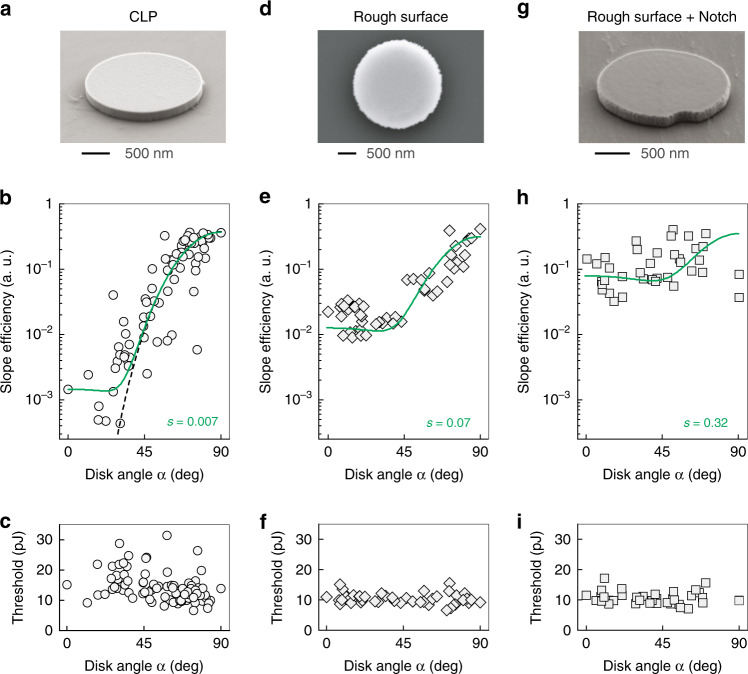
Laser emission from semiconductor microdisks with surface roughness and boundary defects. **a** SEM image of a CLP with smooth sidewalls. **b–c** Slope efficiency (**b**) and threshold (**c**) versus orientation angle *α* of an ensemble of CLPs. Scattering fitting coefficient *s* = 0.007. Black dashed curve: the theoretical prediction for a perfect microdisk. **d** SEM image of a CLP with rough sidewalls. **e–f** Slope efficiency (**e**) and threshold (**f**) versus *α* of CLP ensembles with rough sidewalls. Scattering fitting coefficient *s* = 0.07. **g** SEM image of a notched LP with rough sidewalls. **h–i** Slope efficiency (**h**) and threshold (**i**) versus *α* of notched LP ensembles with rough sidewalls. The lasing wavelengths in (**c**), (**f**), and (**i**) were 1370 ± 10 nm, 1405 ± 10 nm, and 1410 ± 10 nm, respectively. Scattering fitting coefficient *s* = 0.32. Green curves in **b**, **e**, and **h**: best fit based on Eq. (2) in the log scale

To investigate this approach, as a control group, we measured a batch of CLPs with smooth sidewalls (Fig. 3a) embedded in a hydrogel. We measured the output intensity as a function of pump energy *P*_pump_ and obtained the slope efficiency Δ above the threshold for each LP. The orientation angle *α* of each LP was extracted from optical bright-field images by ellipse fitting the LP outline. The output power at each pump level can be readily obtained from the slope efficiency using $P_{{\mathrm{tot}}}\left( \alpha \right) = {\mathrm{{\Delta} }} \cdot (P_{{\mathrm{pump}}} - P_{{\mathrm{th}}})$, where *P*_th_ is the threshold pump energy. If only the output intensity at one specific pump energy was recorded, then the angle dependence analysis would be compounded by the difference in the pump thresholds among the LPs. Figure 3b shows the dependence of the slope efficiency on the orientation angle *α* of 100 microdisks. The fitting parameter *s* = 0.007 obtained with the scattering model (Eq. (2), green curve) reveals that the small imperfections on the LP surface only scatter 0.7% of the output emission on average and result in a small *R* ≈ 0.004 or −24 dB for this CLP ensemble. This value of *R* is likely to be overestimated because Δ values less than 10^−3^ are not reliably measured due to the finite dynamic range of the spectrometer. Lasing thresholds were found to be 13 ± 5 pJ, showing very little dependence on the disk orientation (Fig. 3c). LPs with similar angles exhibit substantial variations in the slope efficiency and threshold. This was attributed to their slightly different sizes causing variations in the laser wavelength across the gain bandwidth, and surface condition.

To increase the surface roughness, we fabricated LPs in the same way as the CLPs but using a different RIE chemistry that produces rougher sidewalls (Materials and methods). This resulted in a batch of LPs with an azimuthal variation of ~50 nm in the disk radius (Fig. 3d). These LPs showed reduced dependence of the slope efficiency on *α* without degrading the lasing threshold, as shown in Fig. 3e, f, respectively. The curve fit yields *s* = 0.07, meaning that the increased surface roughness is responsible for scattering 7% of the light, which results in an improvement in *R* from −24 to −14 dB.

### LPs with defined boundary defects

To further improve upon these results, we artificially introduced sub-wavelength semicylindrical defects with a negative (notch) or positive (bump) curvature. Simulation results illustrate that *P*_tot_(0°), which is almost entirely due to out-of-plane scattering from the boundary defect, increases with increasing defect size (Fig. S3b). When the defect size approaches half of the wavelength in the cavity medium, *P*_sc_(0°) is close to saturation. The in-plane emission is slightly changed by the presence of a bump or notch^23^ (Fig. S3d). In both cases, we found that the boundary defects provide substantial out-of-plane scattering useful for reducing the dynamic range of the slope efficiency.

Experimentally, we induced a notch or a bump with a diameter of 200 nm in microdisks using electron-beam lithography and roughness-inducing RIE processes (Materials and methods). The resulting nanostructure is shown in Fig. 3g and Fig. S4. To confirm the scattering effect of the boundary defect, we first measured these LPs on-chip after partial undercutting to create a supporting pillar. High-resolution maps of the laser emission in the out-of-plane direction show that strong emission is generated in the vicinity of the boundary defects with a peak intensity >1000 counts (Fig. S4d). For comparison, a CLP with rough sidewalls shows a doughnut-like pattern, with emission arising primarily from the disk boundary with a peak intensity of ~ 200 counts (Fig. S4d).

Notched LPs with rough sidewalls (Fig. 3g) were transferred into a hydrogel suspension to investigate their angle dependence. The measured slope efficiency showed a further reduced dependence on *α*, as shown in Fig. 3h. Through fitting with the scattering model, it is found that the combination of surface roughness and a boundary notch scatters approximately 32% (*s* = 0.32) of the lasing light into other directions, resulting in a much improved min-max ratio *R* of 0.27 or −5.7 dB. Notched LPs with similar angles exhibit slightly larger variations in the slope efficiency, which is attributed to the different laser mode numbers due to the non-uniformity in the disk size (Fig. S7c). In addition, since the threshold energy of the semiconductor lasers is mainly determined by the energy required to overcome the optical absorption and to reach transparency, even though the defect slightly reduces the radiation and scattering-limited *Q* factor of the cavity (Fig. S3b), notched LPs have a similar threshold to CLPs, approximately 10±4 pJ, independent of the orientation angle *α* (Fig. 3i).

### **L**Ps coated with a scattering layer (scLPs)

An alternative approach to achieving omnidirectional emission is to incorporate extra-cavity inhomogeneities. This could be realized by coating the microdisks with nanoparticles with a large refractive index but low absorption loss. We chose silicon nanoparticles (SiNPs) due to their high refractive index of 3.48 and nearly zero imaginary part at near-infrared wavelengths. In a three-dimensional (3D) FEM model, SiNPs were randomly placed on top of a microdisk, and a thin silica coating layer was applied. The simulation result confirms a strong light scattering effect of the monolayer of SiNPs (Fig. S5c). Although SiNPs are placed on only one side of the disk, the far-field radiation patterns exhibit considerable emission from both faces, implying that coating of SiNPs on a single microdisk surface is sufficient to improve output omnidirectionality.

**Fig. 4:**
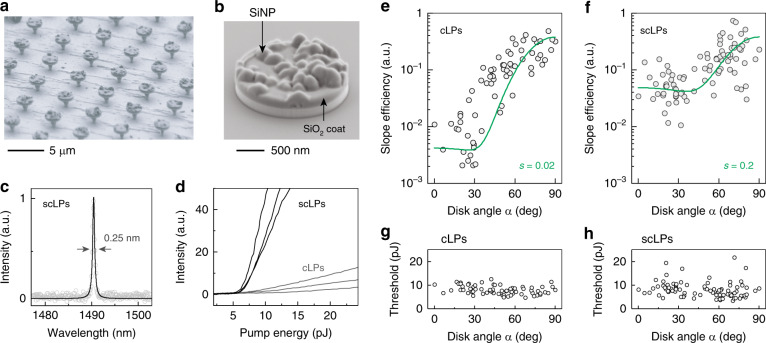
Lasing emission of scLPs. **a** SEM image of an array of scLPs on pillars. **b** SEM image of an scLP after detachment. **c** Typical emission spectrum of an scLP fitted with a Lorentzian lineshape, showing a full width at half maximum of 0.25 nm. **d** Typical output curves versus pump energy for flat cLPs (gray curves) and scLPs (black curves). **e**–**f** Slope efficiency versus orientation angle *α* of cLP and scLP ensembles. Theoretical fits (green curves) yielded scattering coefficients of *s* = 0.02 and *s* = 0.2, respectively. **g**–**h** Lasing threshold of cLP and scLP ensembles as a function of disk angle *α* in a hydrogel

To realize this design, we devised a novel top-down fabrication method (Materials and methods, Fig. S5d) for single-sided coating (Fig. 4a). This technique enabled SiNPs with a size of 30–50 nm to be placed at a distance of ~15 nm from the InGaAsP microcavity by embedding them inside a silica cap attached to the cavity (Fig. 4b). We termed this specific type of OLP a ‘scatterer-coated laser particle’ (scLP). Importantly, unlike the semicircular notch OLPs mentioned earlier, scLPs do not rely on nanometer-scale boundary defects patterned using electron-beam lithography to generate scattered light. Therefore, they are amenable to high-throughput production by UV lithography, rendering them suitable for practical applications.

In this experiment, an InGaAsP wafer with a gain bandwidth of ~1400–1500 nm was used. The output emission of an scLP in a hydrogel typically features a single peak with an FWHM of 0.25 nm (Fig. 4c), similar to that of control LPs (cLPs) that possess a silica cap without embedded SiNPs. The lasing linewidth is determined by the carrier-induced index modulation under pump pulses. The input-output curves in Fig. 4d measured on flat disks show that scLPs have larger slope efficiency than control LPs (the input-output curves plotted on a logarithmic scale are shown in Fig. S5h). cLPs suspended in a hydrogel exhibit *s* = 0.02 and *R* = 0.011 (Fig. 4e). scLPs produce a higher degree of omnidirectionality with a fit parameter of *s* = 0.2 and *R* = 0.13 or −8.8 dB (Fig. 4f). Both scLPs and cLPs have similar threshold distributions (*P*_th _= 8 ± 3 pJ), which show no dependence on the disk orientation (Fig. 4g, h).

### **C**ontinuous cell tracking using OLPs

To demonstrate the detection reliability of omnidirectionally emitting LPs for cellular tracking, ~10^5^ OLPs (scLPs) and CLPs (cLPs) were fabricated and separately transferred into cell media for HeLa cell coculture (Materials and methods). Within 12 h of incubation in vitro, both CLPs and OLPs were efficiently internalized by cells through the non-specific process of macropinocytosis^6,7,32,33^. The orientation of loaded LPs was observed to vary continuously within the cells, resulting in random disk orientations at any given moment (Supplementary video).

**Fig. 5:**
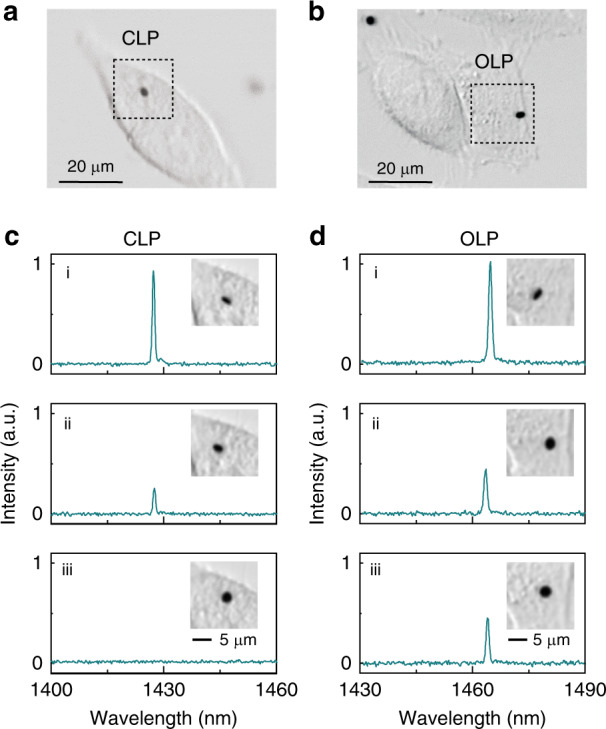
Lasing emission of OLPs in live cells. **a**, **b** CLP (**a**) and OLP (**b**) inside live HeLa cells. **c** Output spectra versus orientation of a CLP in a live HeLa cell. The spectra were acquired at the same time as the optical images in the insets (i–iii). **d** Output spectra versus orientation of an OLP in a live HeLa cell. The spectra were acquired at the same time as the optical images in the insets (i–iii)

Using a custom-modified confocal microscope^7^, bright-field images and lasing emission of LPs in cells were obtained as ground-truth data for tracking CLPs and OLPs in live HeLa cells (Fig. 5a, b). Figure 5c, d show output spectra at three time points from a CLP and an OLP, respectively, along with the disk orientations. In each case, the LPs were pumped under the same conditions. For both the CLP and OLP, strong emissions are observed from the disks when seen edge-on, as expected (Fig. 5c-i and d-i). However, for flat disks (Fig. 5c-iii and d-iii), no laser peak is detected for the CLP, while a distinct lasing peak is observed for the OLP, with only a 50% reduction in power compared to the edge-on case.

**Fig. 6:**
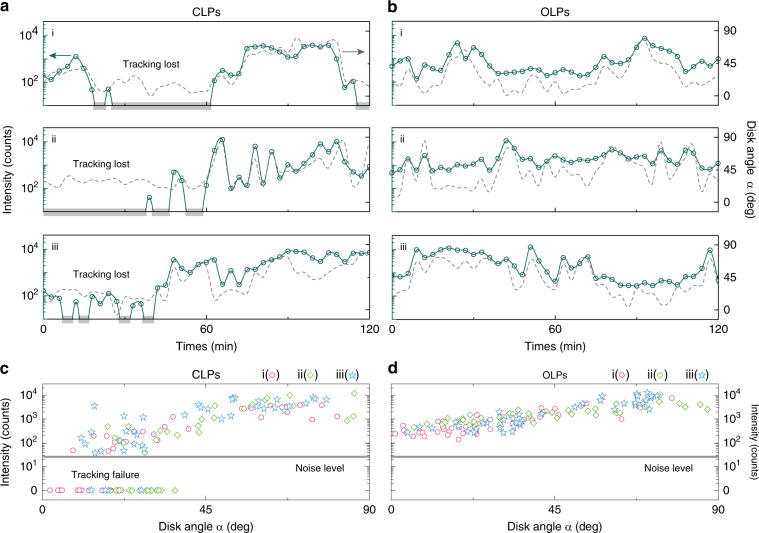
OLPs for continuous single-cell tracking. **a**, **b** Lasing intensity (points) and orientation angle (dashed) traces as a function of time for three tracked CLPs (**a**) and OLPs (**b**) internalized by cells. For low orientation angles, the signal received from CLPs falls below the detection threshold, and the tracking is lost. **c**, **d** Lasing intensity versus disk angle *α*

Next, we acquired time-lapse maps of several LPs of each type over a period of 2 h. Because of the random-walk movement of LPs inside the cytoplasm, we scanned the focal plane in the z-direction to obtain Z-stack lasing maps and bright-field images every 3 min for 2 h. Since LPs have single-mode emission with sub-nanometer linewidth (Fig. S2a), each LP was identified and tracked over time using a clustering algorithm based on the positional and spectral traces. For each LP, the integrated intensity of the lasing peak and orientation angle *α* were extracted from all spectral frames and bright-field images associated with the disk, respectively. Figure 6a, b show the time-lapse intensity traces of three typical CLPs and three OLPs, respectively. Whenever the orientation angle *α* became small (i.e., flat disks), the lasing peak intensities of the CLPs were overwhelmed by background noise (~30 counts in our experiment), leading to frequent optical reading failure in cell tracking. In the 2-h traces, the signal was lost in 28% of the frames (Fig. 6c). By contrast, OLPs could be continuously tracked over the entire 2 h even when they rotated to small *α* (Fig. 6b, d).

## Discussion

In conclusion, we have demonstrated highly omnidirectional LPs with a min-max ratio *R* ≈ −5.7 dB, a low threshold, a narrow linewidth, and single-mode lasing, which enable reliable cell tagging and continuous cell tracking. We expect OLPs to enhance the tracking reliability in applications including deep tissue imaging, where intrinsic tissue scattering does not overcome the low signal collection efficiency for flat disks (Supplementary Note 4). The scattering elements introduced at the boundary and flat surface were effective, directing up to 20-32% (*s* = 0.20–0.32) of the collected laser emission to all directions.

To enable long-term operation in aqueous biological environments, semiconductor LPs may need an additional protective layer^7,34^. The simulated results in Fig. S7 reveal that *P*_tot_(*α*) remains highly omnidirectional for OLPs with a protective silica coating layer. The notched OLP design could be improved by incorporating more than one boundary defect into the cavity design (Fig. S3f). Since state-of-the-art optical lithography offers a resolution of better than 150 nm, this method has the potential for low-cost, high-volume production of OLPs. scLPs are readily mass producible using optical lithography.

The high-brightness omnidirectional emission significantly improves the SNR of LPs, resulting in reliable spectral identification and spatial tracking without increasing the exposure time. OLPs allow for continuous and high-speed tracking of single cells, which, combined with the massive spectral multiplexing capability of LPs^7^, enables the study of cellular heterogeneity at the single-cell level in large-scale 3D biological specimens. In addition to cell tracking, the omnidirectionality will facilitate other applications of LPs, such as cellular and biochemical sensing and single-cell analysis in microfluidics^2,8,14,35–37^, by ensuring a high SNR.

## Materials and methods

### Fabrication and transfer of LPs

Microdisk resonators were fabricated starting from epitaxially grown III–V semiconductor wafers consisting of a 300-nm-thick buffer layer of undoped InP, a 200-nm-thick active layer of InGaAsP, and a 100-nm-thick capping layer of undoped InP over an InP substrate.

Defect LPs by e-beam lithography: Microdisk lasers with nanoscale protrusions or indentations were fabricated on semiconductor wafers. The patterns were defined by 100 keV electron-beam lithography (JBX6300FS, JEOL) on a negative-tone resist (SU8, 50% dilution) and transferred to the semiconductor by reactive-ion etching (Oxford Plasmalab 100 ICP) using a mixture of chlorine and argon. The remaining resist was removed by oxygen and fluoroform plasma treatment and ultrasonic agitation in an N-methyl-2-pyrrolidone-based organic solvent at elevated temperatures (Microposit Remover 1165, Dow Chemicals). Corresponding control samples with circular shapes were fabricated by the same method. For the on-pillar disks, the supporting pillars were undercut by wet chemical etching in diluted HCl. During this last step, the capping layer was also removed. Samples with intentional surface roughness were fabricated following a similar process flow but using hydrogen bromide chemistry during RIE.

Control LPs (cLPs) and scLPs by optical lithography: First, the InP capping layer was removed by etching in 3:1 HCl:H_2_O for 10 s. Cleaning of the surface was then performed using acetone, isopropyl alcohol (IPA) and water followed by O_2_ plasma (30 s, 100 W, 40 sccm O_2_) (SCE 106, Anatec Ltd). Next, 15 nm of SiO_2_ was deposited by plasma-enhanced chemical vapor deposition (PECVD) (Surface Technology Systems). Silicon nanoparticles (30–50 nm, US Research Nanomaterials, Inc.) in IPA were filtered using a centrifuge filter (pore size: 450 nm), and particle aggregations were broken up by a probe sonicator (Fisher Scientific). Immediately before spinning the silicon nanoparticles, the individual chips were cleaned using O_2_ plasma (60 s, PE-25, Plasma Etch Inc.). The newly deposited silica layer was wetted with IPA and spread uniformly across the chip at a spin speed of 2000 rpm for 45 s (Laurell Technologies Corporation). Twenty microlitres of Si nanoparticles suspended in IPA was dynamically dispensed at 600 rpm before increasing the spin speed to 3000 rpm, where it was held for 120 s, during which the IPA fully dried. During this spin-coating step, silicon nanoparticle aggregates approximately a hundred nanometers in scale formed on the wafer surface (Fig. S5e).

Next, a second 250-nm-thick layer of SiO_2_ was deposited by PECVD to fully incorporate the nanoparticles into the silica shell. The surface was then cleaned using O_2_ plasma (120 s, Matrix 105). To enhance the photoresist adhesion to the SiO_2_ film, an adhesion promoter (Omnicoat MicroChem) was used before spin-coating (Headway Research, Inc.) the surface with a 3 μm-thick layer of photoresist (SU8-2002 MicroChem). Soft baking procedures followed the manufacturer’s guidelines. Then, 2.5 μm-diameter circles of SU8 photoresist (density: ~3.2 million/cm^2^) were defined using a projection exposure tool (MLA150, Heidelberg Instruments) at a dose of 1500 mJ/cm^2^ at a wavelength of 375 nm. A two-step postexposure bake was used, consisting of 60 s at 65 °C followed by 180 s at 95 °C on a contact hotplate. The resist was developed for 60 s in SU8 Developer (MicroChem). To smooth the sidewalls of the resist and harden it for dry etching, a further bake at 190 °C for 10 min was performed on a contact hotplate. The residual photoresist was removed using a 90 s descum at 100 W and 40 sccm O_2_ (SCE 106, Anatec Ltd). Next, inductively coupled reactive-ion etching (ICP-RIE) using a fluorine-based chemistry was performed (Surface Technology Systems) to define columns consisting of Si nanoparticles embedded in the silica film. Any remaining SU8 resist was subsequently removed using O_2_ plasma ashing (Matrix 105) for 10 min at 220 °C. The Si/SiO_2_ columns were used as a hard mask for a III–V ICP-RIE process that etched a depth of ~1 μm using a chlorine-based chemistry (PlasmaPro 100 Cobra 300, Oxford Instruments).

The corresponding control samples with a silica-capping layer were prepared with the same method without spinning the silicon nanoparticles. To completely detach the microdisks, the substrates were wet-etched face down in 3:1 HCl:H_2_O solution inside a 1 μm pore centrifuge filter for 30 s and filtered thoroughly by at least three repeated cycles of centrifugation and resuspension (via ultrasonication) using ultrapure water.

### Optical characterization

For optical characterization and imaging of microdisks, a laser-scanning LASE microscope modified from a commercial confocal microscope (Olympus FV3000) was used. A pump laser (Spectra Physics VGEN-ISP-POD, 1060–1070 nm, pulse duration 3 ns, repetition rate 2 MHz), with the output power controlled by an acousto-optic modulator and measured by an external photodetector, was coupled to a side port of the laser-scanning unit of the microscope. The day-to-day variation in the measurement of the absolute pump power was up to 30%. The emission from microdisks was collected from the same port and relayed by a dichroic mirror to a NIR spectrometer using an InGaAs linescan camera (Sensor Unlimited 2048 L). A 100 lines/mm grating (0.6-nm resolution over 1150–1600 nm, exposure time: 0.1 ms) was used for threshold characterization, and a 500 lines/mm grating was used for high-resolution linewidth characterization (0.2-nm resolution, 150-nm span, exposure time: 0.1 ms). In both cases, a NIR-optimized, 20X, 0.45-NA objective (Olympus IMS LCPLN20XIR) was used. The high-resolution lasing mapping images were acquired with a 100X, 0.85-NA objective (Olympus IMS LCPLN100XIR) and the NIR spectrometer with the 100 lines/mm grating (0.6-nm resolution over 1150–1600 nm, exposure time: 0.1 ms).

### Numerical simulation of the far-field pattern

The modeling of the passive microdisk resonance was conducted via a series of three-dimensional finite-element simulations (COMSOL Multiphysics 5.3a). We set the refractive indices of the microdisk and hydrogel to 3.445 and 1.334, respectively. The optical absorption of the bulk semiconductor material and laser gain were not considered here. The shape and size of the disk agree with those of the semiconductor laser particles we used in our experiment (obtained by SEM). The thickness of the disk is 200 nm, the diameter of defect LPs and the corresponding control LPs made by e-beam lithography is 2 μm, and the diameter of scLPs and the corresponding control LPs is 2.5 μm. The simulation region was set with perfectly matched layer boundary conditions in all directions. The distance to the perfectly matched layer boundaries as well as the meshing size was chosen after a series of convergence tests. We used the eigenfrequency study (Physics: Radio Frequency, frequency domain) and the far-field domain plug-in to calculate the far-field pattern of the WGM. The randomly distributed nanoparticles for scLPs were generated by an application-builder module. The calculated intensity pattern ($\left| {E_{{\mathrm{far}}}(\theta ,\phi )} \right|^2$) was exported, and a MATLAB script was used to calculate the output pattern through integration over the acceptance solid angle ${\Omega} = 2\pi \left[ {1 - \cos \left( {{\mathrm{asin}}\left( {{\mathrm{NA}}/n} \right)} \right)} \right] = 0.37$ steradians (Eq. (1)). Finally, the output pattern *P*_tot_(*α*) was normalized by the total output energy.

### Cell-culture experiments

HeLa human cervical cancer cells (ATCC) were cultured and maintained with Dulbecco’s modified Eagle medium (DMEM) supplemented with 10% (v/v) fetal bovine serum (FBS) and 1% (v/v) penicillin-streptomycin. Cells were seeded in 8-well chambered glass dishes (Cellvis) at a density of 15,000 cells/cm^2^. After 24 h, 60,000 scLPs were added to one of the culture wells, and 60,000 control LPs were added to a control well, along with the requisite quantity of 10× PBS to ensure isotonicity of the final solutions. After 1 h, the cell media was aspirated and replaced with a fresh volume. The cells were then incubated (Thermo Scientific Heracell 240i) with the LPs at 37 °C and 5% CO_2_ for 8 h to give sufficient time for LP uptake. During imaging, cells were incubated using a microscope stage top incubator (Tokai Hit).

Cell tracking experiments were performed by acquiring time-lapse data every 3 min over a total period of 2 h. During the measurement, cells were placed in a temperature-controlled cell-culture incubator. Six regions were defined: three from the scLP well and three from the control well. Each region, consisting of 320 × 320 × 7 voxels corresponding to a volume of 212 × 212 × 21 μm^3^, was scanned with a NIR pump laser (Spectra Physics VGEN-ISP-POD, pulse duration 3 ns, repetition rate 2 MHz, pulse energy 160 pJ) using a pixel dwell time of 20 μs. Bright-field images were recorded simultaneously with the acquisition of spectral LP emission. For each microdisk, the integrated intensity of the lasing peak was calculated for all spectral frames associated with the disk. Orientations were obtained by analyzing the bright-field images using ImageJ, and the orientation angle was then associated with the maximum integrated intensity of the lasing peak of each disk.

Fluorescence imaging was realized by washing the cells three times with PBS, followed by fixation with 4% paraformaldehyde/PBS (Fisher Scientific), permeabilization with 0.1 Triton X-100/PBS (Fisher Scientific) and incubation with Alexa Fluor 594-Phalloidin (Thermo Fisher) for actin staining and NucBlue Fixed Cell Stain (Thermo Fisher Scientific) for nuclear staining, following the manufacturer’s guidelines.

## Supplementary information

Supplementary information

Supplementary video
