# Author Correction: Laser particles with omnidirectional emission for cell tracking

**DOI:** 10.1038/s41377-021-00481-1

**Published:** 2021-02-26

**Authors:** Shui-Jing Tang, Paul H. Dannenberg, Andreas C. Liapis, Nicola Martino, Yue Zhuo, Yun-Feng Xiao, Seok-Hyun Yun

**Affiliations:** 1 Harvard Medical School and Wellman Center for Photomedicine, Massachusetts General Hospital, Boston, MA USA; 2 State Key Laboratory for Mesoscopic Physics and Frontiers Science Center for Nano-optoelectronics, School of Physics, Peking University, 100871 Beijing, China; 3 Harvard-MIT Health Sciences and Technology, Massachusetts Institute of Technology, Cambridge, MA USA

**Keywords:** Solid-state lasers, Imaging and sensing

Correction to: *Light: Science & Applications*

10.1038/s41377-021-00466-0 published online 25 January 2021

We would like to correct the SEM image of Fig. 3a on page 5.

**Fig. C1:**
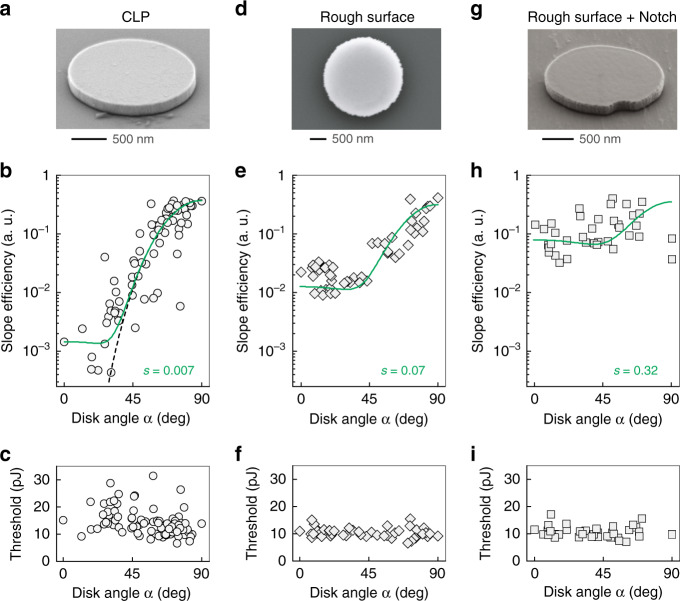
The figure a in this figure is the corrected version for Fig. 3a in the original article.

The SEM image of Fig. 3a in the original article should be corrected to the Fig. C1a below.

We would like to apologize for any inconvenience this may have caused.

