# Supplementary material for: Laser particles with omnidirectional emission for cell tracking
Source: Light Sci Appl. 2021 Jan 25;10:23. doi: 10.1038/s41377-021-00466-0 (PMC7835369; doi:10.1038/s41377-021-00466-0)
Supplement: Supplementary file 1 — Supplementary information [file 41377_2021_466_MOESM1_ESM.pdf]

SUPPLEMENTARY INFORMATION

Laser particles with omnidirectional emission for cell tracking

Shui-Jing Tang,[1,2] Paul H. Dannenberg,[1,3] Andreas C. Liapis,[1]
Nicola Martino,[1] Yue Zhuo,[1] Yun-Feng Xiao,[2] and Seok-Hyun Yun,[1,3]

[1]Harvard Medical School and Wellman Center for Photomedicine,
Massachusetts General Hospital, Boston, Massachusetts, United States.
[2]State Key Laboratory for Mesoscopic Physics and Frontiers Science Center for
Nano-optoelectronics, School of Physics, Peking University, Beijing 100871, China.
[3]Harvard-MIT Health Sciences and Technology, Massachusetts
Institute of Technology, Cambridge, Massachusetts, United States.

Correspondence: Seok-Hyun Yun (syun@hms.harvard.edu) or Yun-Feng Xiao (yfxiao@pku.edu.cn).
These authors contributed equally: Shui-Jing Tang, Paul H. Dannenberg, Andreas C. Liapis.

Supplementary Figures.

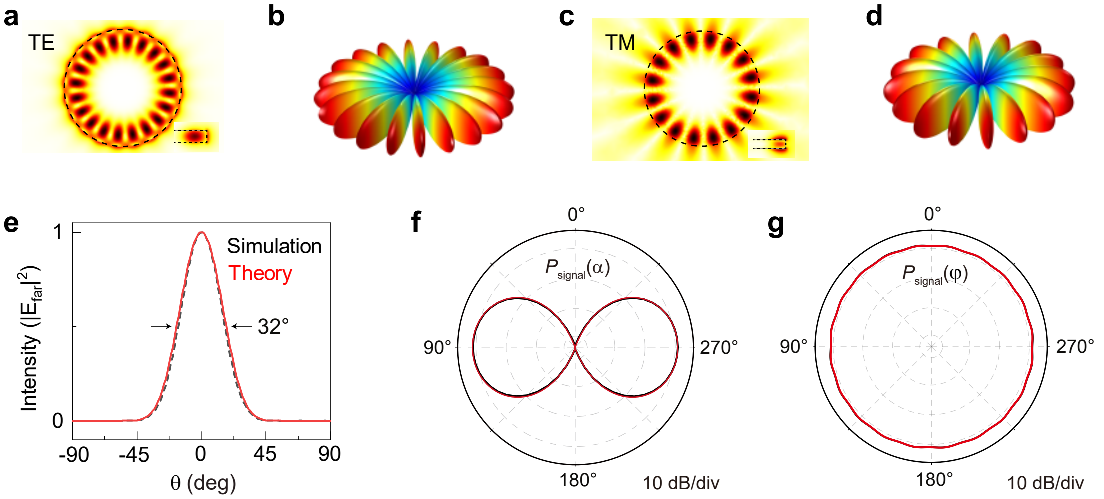

Fig. S1: Radiation patterns of whispering gallery modes of a microdisk in water. a-d, Electric-field distributions $|E|$ and far-field radiation patterns $|E_{\text{far}}|$ of the TE (a, b) and TM (c, d) modes. Insets of a and c: Cross-sections of the electric-field distributions of the TE and TM modes. The radiation-limited Q factors of the TE and TM modes are 11,500 and 113, respectively. **e**, The far-field intensity distribution of the TE mode as a function of the polar angle, calculated by 3D FEM simulation (black dashed curve) and by scalar diffraction theory (red solid curve). The divergence angle (full width at half maximum, FWHM) is about 32°. **f-g**, The power P_{signal} collected by an objective lens with a finite numerical aperture (NA = 0.6) for different orientations of the disk showing a pronounced dependence on the tilt angle α (f) but not on the azimuth angle φ (g). (black: 3D FEM simulation; red: scalar diffraction theory).

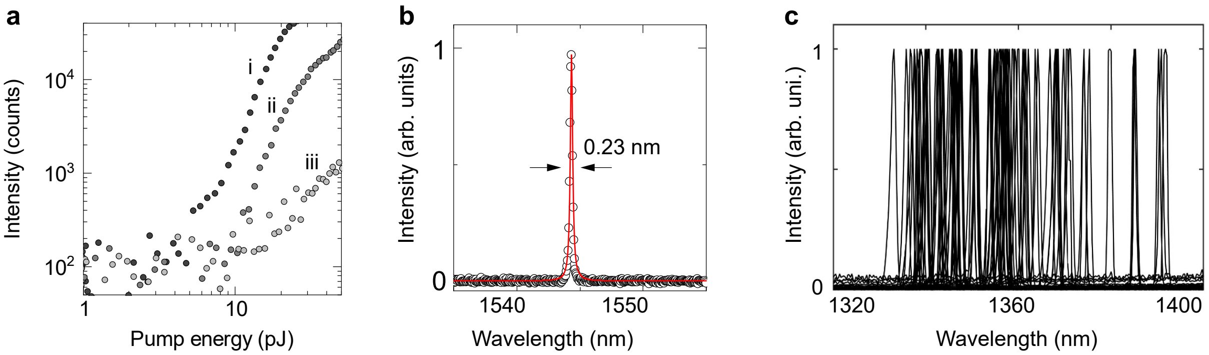

Fig. S2: Lasing threshold and linewidth of conventional LPs. a, P_{signal} versus pump energy P_{pump} on a logarithmic scale of CLPs suspended in hydrogel with different orientations in Fig. 1e. The lasing wavelength of these samples ranges in 1230-1310 nm. **b**, Typical single-mode emission spectrum of a microdisk (made from a different wafer with a gain bandwidth at 1500-1600 nm). Red curve: fit with a Lorentzian line-shape with a FWHM of 0.23 nm. **c**, Normalized lasing emission spectra of a batch of conventional LPs made from another wafer with a different gain bandwidth.

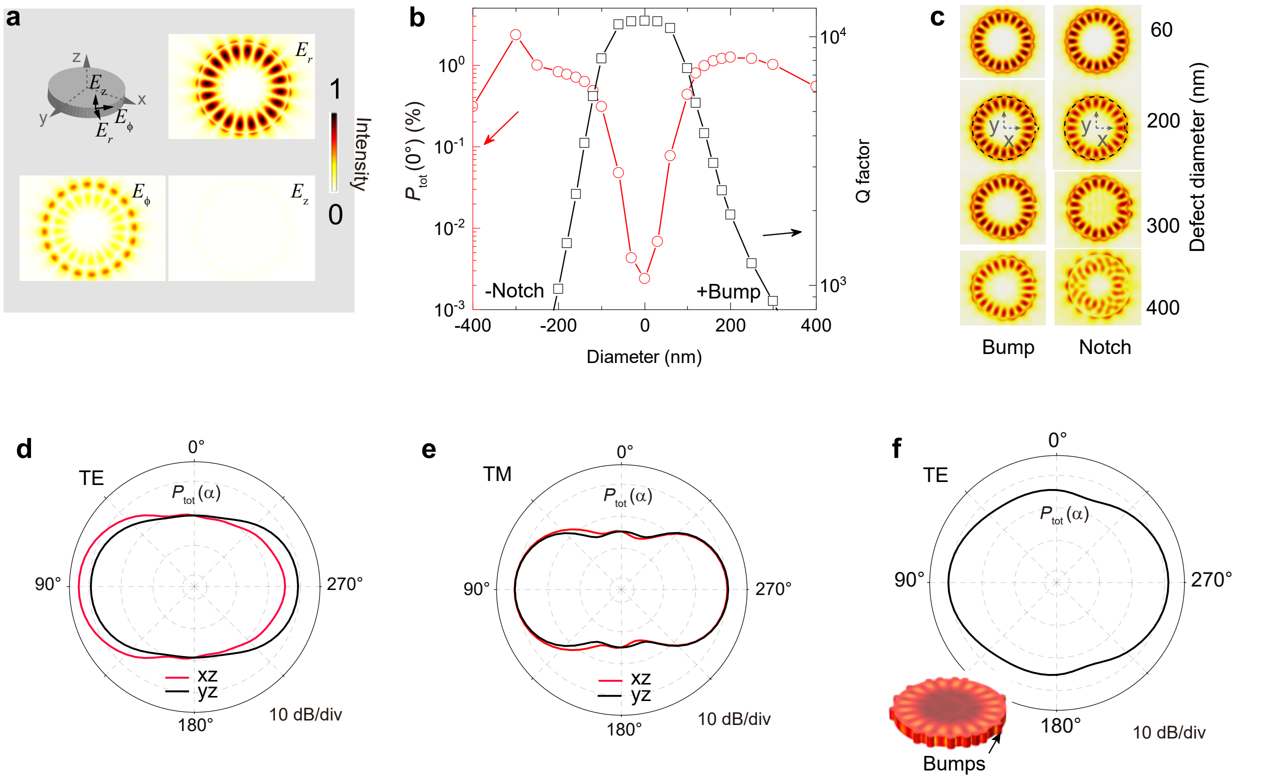

Fig. S3: Numerical simulation of OLPs with a boundary defect. **a**, Electric-field distribution of the TE mode of a conventional LP. **b**, $P_{\text{tot}}(0^\circ)$ and Q factor of an OLP with a boundary defect as functions of the defect size. Positive diameters correspond to a protrusion (bump) and negative to an indentation (notch). **c**, Electric-field distributions of a TE mode of a microdisk versus defect size. **d-e**, Simulated $P_{\text{tot}}(\alpha)$ for a microdisk with a 200-nm-diameter hemicylindrical boundary notch (d, TE mode; e, TM mode). **f**, Mode pattern (inset) and simulated $P_{\text{tot}}(\alpha)$ of an OLP with 15 bumps each with a diameter of 140 nm placed randomly on the boundary of disk. The corresponding radiation and scattering limited Q factor is 1,200.

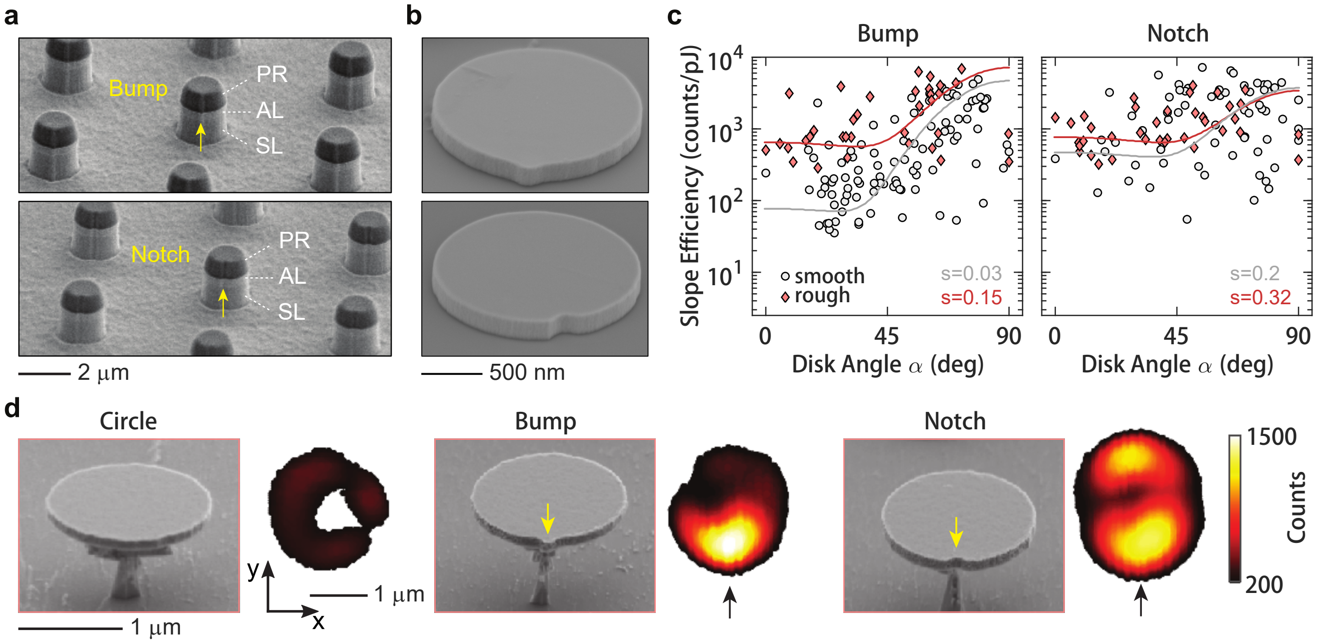

Fig. S4: Lasing emission of LPs with boundary defects. **a**, SEM of arrays of bump and notch LPs after the reactive ion etching step, showing the photoresist (PR), active layer (AL) and substrate layer (SL). **b**, SEM of bumped and notched disks after detachment. **c**, Slope efficiency versus tilt angle α of bumped and notched disks with smooth (circles) and rough (diamonds) sidewalls. **d**, SEM and lasing-intensity maps of circular, bumped, and notched disks with rough sidewalls on support pillars at the same pump energy (at 100 μs exposure time per pixel). The NA of the objective lens is 0.45 in c and 0.85 in d.

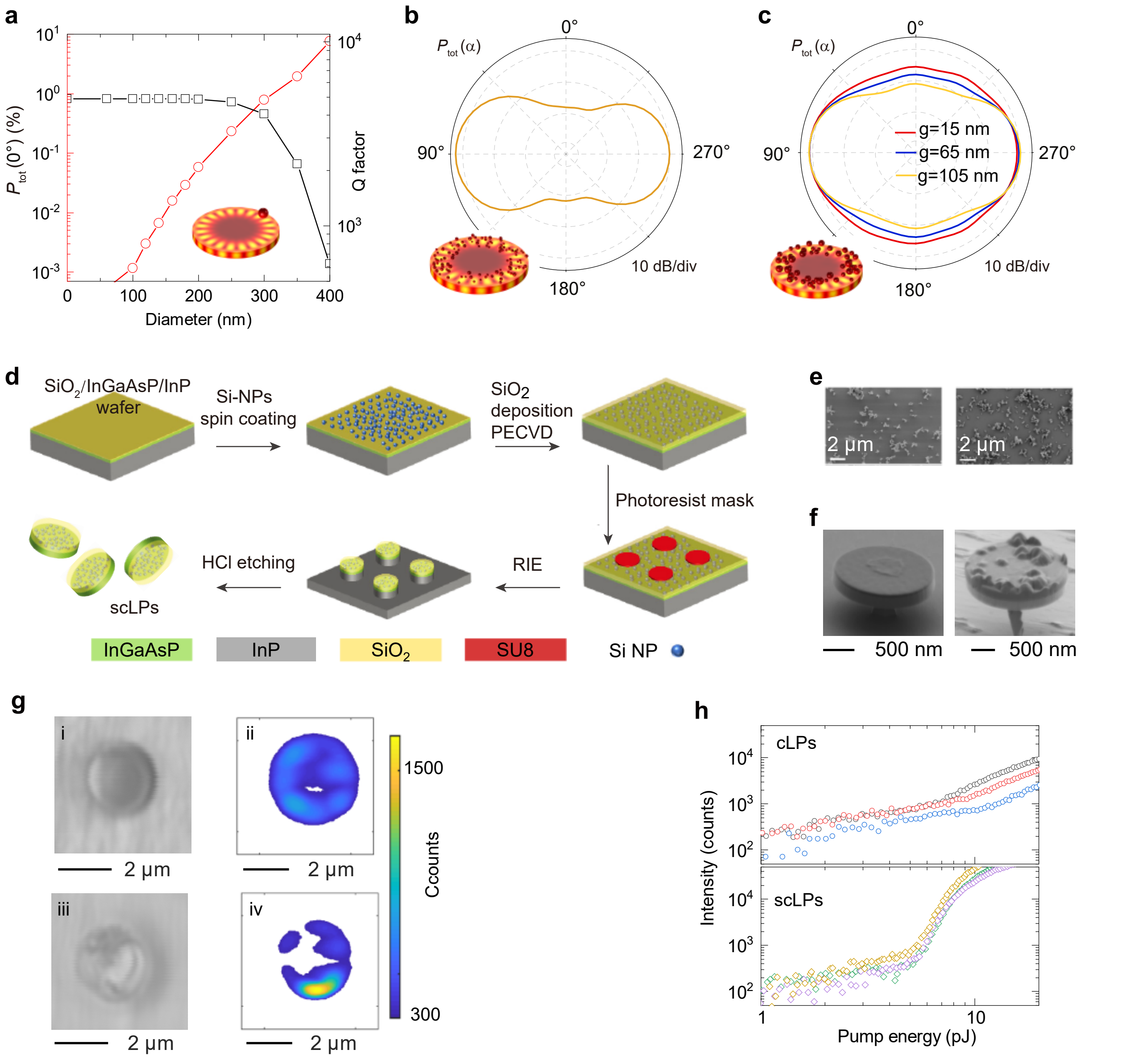

Fig. S5: Numerical simulation and optical characterization of scLPs. **a**, $P_{\rm tot}(0°)$ and Q factor for a microdisk with a single silicon nanoparticle (SiNP) resting on its surface as functions of the particle size. Here, the diameter of disk is 2.5 μm and the distance between the SiNP and the disk is 15 nm. Inset: the simulation model. **b**, Simulated $P_{\rm tot}(\alpha)$ of a scLP with 100 small SiNPs (diameters: 50–100 nm) embedded in a silica capping layer (thickness: 250 nm), with a SiNP-disk gap distance of 15 nm. The radiation-scattering limited Q factor of the resonance mode is 4,500. **c**, Simulated $P_{\rm tot}(\alpha)$ of a scLP with 50 large SiNPs (diameters: 100–200 nm) embedded in a silica capping layer (thickness: 250 nm), with a different SiNP-disk gap distance: g=15 nm (red), 65 nm (blue), and 105 nm (yellow). The radiation-scattering limited Q factors of corresponding resonance mode are 2,600 (g=15 nm), 3,700 (g=65 nm), 4,300 (g=105 nm) respectively. **d**, Fabrication process of scLPs. The separation between the disks and the nanoparticle layer is controlled by the thickness of the initial SiO_2 layer deposited by PECVD. **e**, Density control of SiNPs spin-coated on an InGaAsP wafer. **f**, SEM of a control LP (left) and a scLP (right) on pillars. **g**, Optical images and corresponding lasing-intensity maps of a control LP (i, ii) and a scLP (iii, iv). The lasing-intensity maps of both control LP and scLP were obtained at the same pump energy. **h**, The output emission versus pump energy on a logarithmic scale for three representative flat ($\alpha = 0$) cLPs (top) and scLPs (bottom) in Fig. 4d.

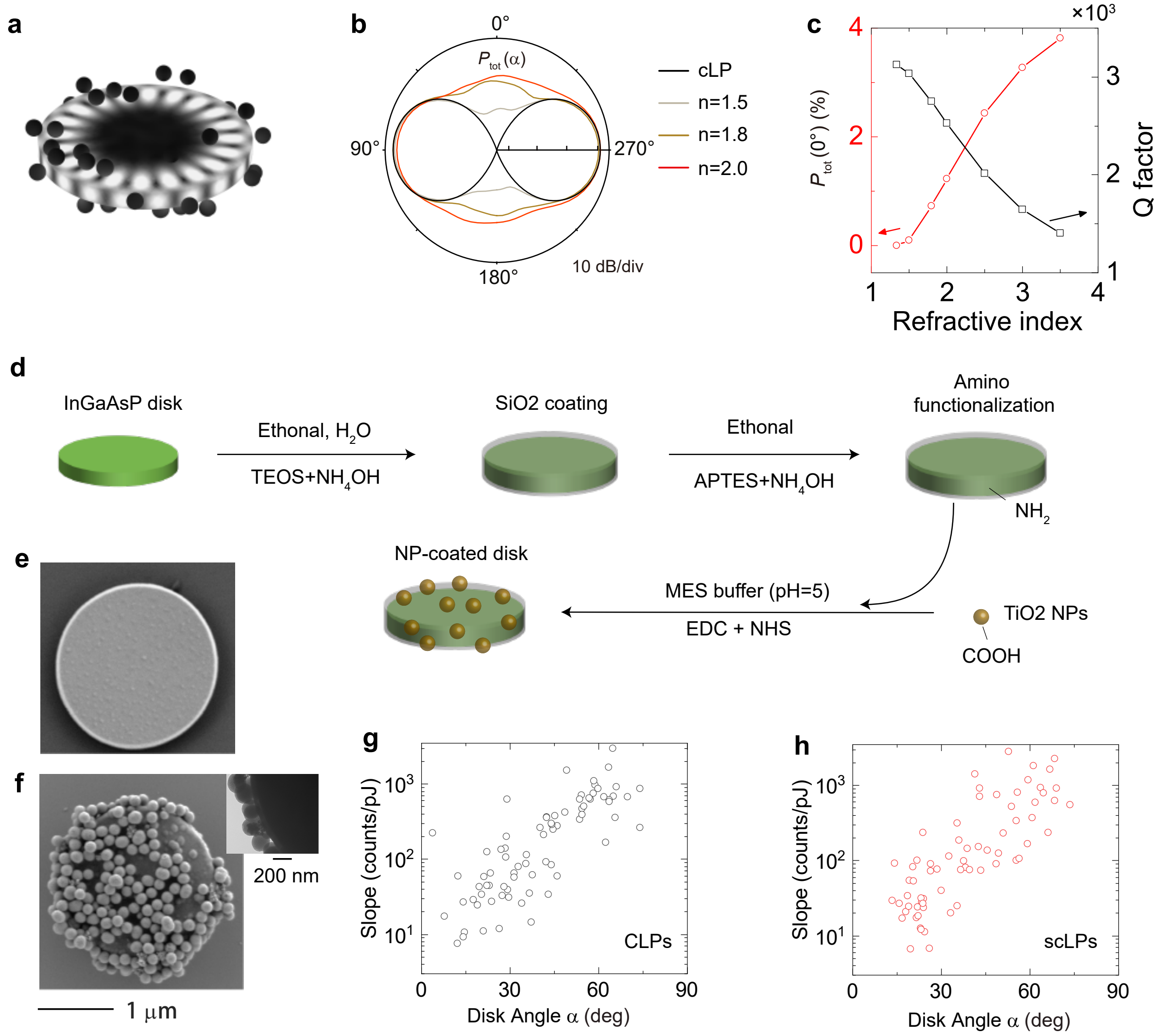

Fig. S6: Lasing emission of scLPs obtained by chemical functionalization. **a**, Simulated model of a scLP in which 47 spherical nanoparticles (diameter: 200 nm) are randomly distributed around the disk. The gap distance between the disk and nanoparticles is 50 nm with a refractive index of 1.40, and the refractive index of environment is 1.334. **b**, Simulated $P_{\mathrm{tot}}(\alpha)$ when the refractive index of the nanoparticles is 1.334, 1.5, 1.8 and 2.0. **c**, $P_{\mathrm{tot}}(0°)$ and radiation-scattering limited Q factor versus the refractive index of the nanoparticles. **d**, Chemical functionalization of scLPs. **e**, SEM of a conventional LP. **f**, SEM and TEM (inset) of a scLP covered with 200-nm-diameter TiO_2 nanoparticles. **g-h**, Slope efficiency versus orientation angle α of CLPs and scLPs suspended in hydrogel.

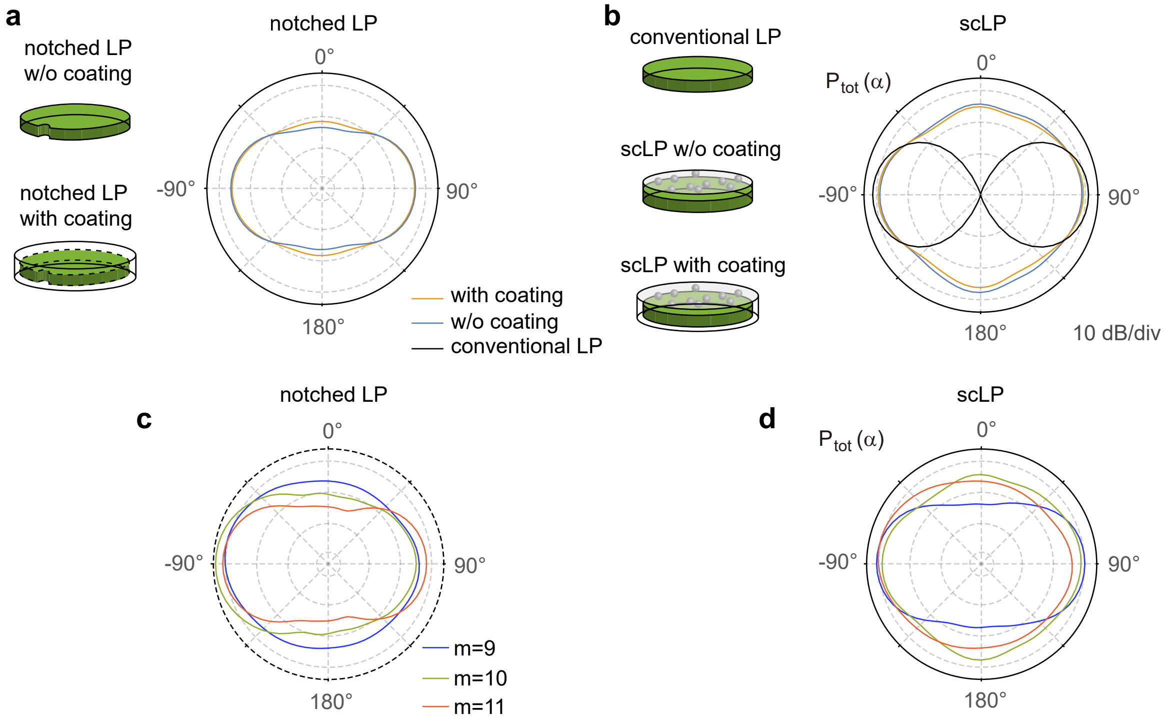

Fig. S7: Simulated $P_{\mathbf{tot}}(\alpha)$ of omnidirectional LPs considering the protective silica coating and different mode number. **a**, Semiconductor microdisk with a 200-nm-dameter semicylindrical notch on the boundary. **b**, Semiconductor microdisk coated with a scattering layer (scLP) in which silicon nanoparticles (100-200 nm diameter) are embedded in a 250-nm-thickness capping silica layer. The thickness of the protective silica coating is 250 nm. The refractive indexes of the semiconductor microdisk, silicon nanoparticles, silica coating and the surrounding environment are 3.45, 3.48, 1.46 and 1.33 respectively. The radiation-scattering limited Q factors: (a) 1,000 for notched LP without silica coating and 1,400 for LP with silica coating; (b) 2,400 for scLP without silica coating and 1,900 for scLP with silica coating. **c-d**, Simulated $P_{\text{tot}}(\alpha)$ of notched LP (c) and scLP (d) for cavity modes with different azimuth mode number.

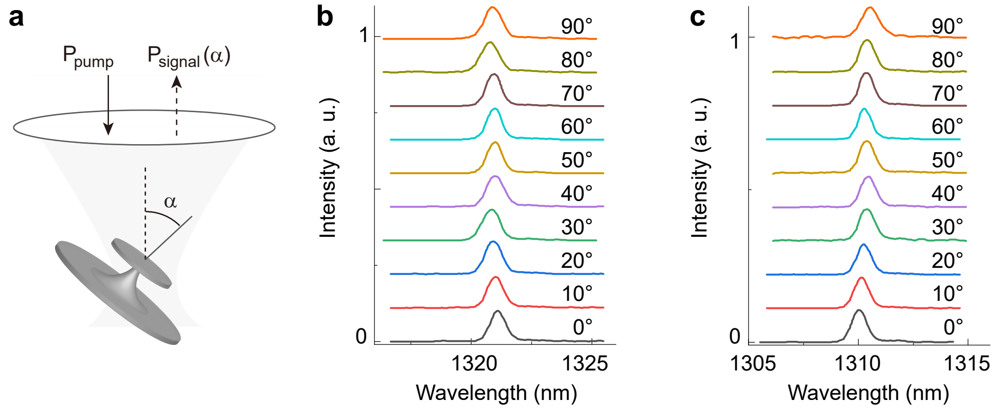

Fig. S8: Laser mode position versus tilt angle of microdisk LPs **a**, A schematic of the experimental setup. **b-c**, The lasing spectra versus the title angle of two representative microdisk LPs on supporting pillars.

Supplementary Notes

Supplementary Note 1: Far-field modeling of WGM resonance

A scalar diffraction theory of light emanating from a microdisk of radius R into the far-field has been well developed in Ref. [1] and is given in the spherical coordinates (r, θ, ϕ), as:

$$|E_{\text{far}}(\theta,\phi)|^2 \propto \frac{\cos^2\phi}{r^2}\left|\frac{F(k\cos\theta)}{H_m^{(2)}(kR\sin\phi)}\right|^2, \tag{S1}$$

where $k = n\omega/c$ is the wave number, ω is the angular frequency, n is the refractive index of external environment of the microdisk, F is the Fourier transform of the near-field distribution $f(z)$ on the cylindrical surface $\rho = R$ in cylindrical coordinate (ρ, θ, z), and $H_m^{(2)}$ is the Hankel function of the second kind.

Considering a typical semiconductor microdisk laser (refractive index 3.445, radius $R = 1\,\mu$m, and thickness of 200 nm), the m=10th-order TE WGM at the resonant wavelength of 1270 nm is obtained by a 3D FEM simulation (Fig. S1a). For simplicity, the near-field distribution $f(z)$ is approximated by a Gaussian function with a FWHM equal to the disk thickness 200 nm. The calculated far-field intensity in Fig. S1e illustrates that its divergence angle (i.e. FWHM) is about 32°. The theoretical result is in good agreement with the result calculated by 3D FEM simulation, and can be approximated by $|E_{\text{far}}(\theta)|^2 \propto \sin^{18}(\theta)$ (Figs. S9a). The deviation when the normalized $|E_{\text{far}}(\theta)|^2 < 10^{-5}$ is attributed to the limited accuracy in numerical simulation or the Gaussian approximation of near-field distribution.

Experimentally, the output emission is collected by a lens with a finite numerical aperture (NA). The power collected from a microdisk that is tilted with respect to the viewing axis by an angle α is given by

$$P_{\text{signal}}(\alpha, \text{NA}) \propto \int_\Omega |E_{\text{far}}(\theta,\phi)|^2 \sin\theta\, \mathrm{d}\theta\, \mathrm{d}\phi, \tag{S2}$$

where the integration is performed over the solid angle Ω defined by a cone with half angle asin(NA/n) centered on α. Figure S1f shows the dependence of P_{signal} on the tilted angle α of the disk (Fig. S1f). The theoretical result of P_{signal} is in good agreement with the result calculated by 3D FEM simulation, which could be approximated by $P_{\text{signal}} \propto \sin^{10}(\alpha)$ for NA = 0.45 (Fig. S9b). Note that, when standing waves are formed by two counter propagating WGMs, the lobed azimuthal structure in the far-field intensity profile [2] is approximately averaged out provided the collection NA is greater than $0.5\pi n/m$, or > 0.2 for m=10 (Fig. S1g).

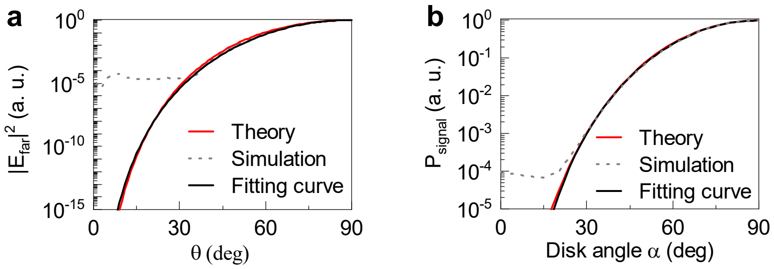

Fig. S9: Far-field radiation pattern $|E_{\text{far}}(\theta)|^2$ (a) and the collected output emission $P_{\text{signal}}(\alpha)$ (b). Red solid curves: the scalar diffraction theory; Gray dashed curves: 3D FEM simulation. Black solid curve: the fitting curves of $|E_{\text{far}}(\theta)|^2 = \sin^{18}(\theta)$ (a) and $P_{\text{signal}}(\alpha) = \sin^{10}(\alpha)$ (b).

Supplementary Note 2: The ratio R of the minimum and maximum intensities as a function of NA

To quantify the angle dependence of P_{signal}, we calculate the ratio R of the minimum and maximum collected powers from the result of Eq. S2. Note that R is a strong function of the NA of light collection. Figure S10 describes the dependence of R for a conventional LP as a function of NA/n . For high NA > 0.7, we find $R \approx$110 (NA/n-1) dB.

In this work, we consider a LP with R > 0.01 as being omnidirectional, and R > 0.1 as highly omnidirectional. These conditions could be achieved with a CLP when NA > 1.1 and NA > 1.2, respectively. Using such high NA, however, may not be allowed in certain instruments, nor be a desirable solution because its diffraction-limited volume at the focus would become much smaller than the size of an LP, reducing the collection efficiency and causing intensity variations depending on the focal position. For LPs with a size of D and wavelength of λ, the maximum NA without significantly losing collection efficiency is $\sim \lambda/D$. For $D = 2\ \mu$m and λ = 1270 nm, the collection NA should be less

than 0.64. As heuristic approximates, for NA < 0.7 we find $P_{\text{signal}}(\alpha, \text{NA}) \propto |\sin\alpha|^q$, where $q \approx 18 - 18$*NA and $R < -60$ dB.

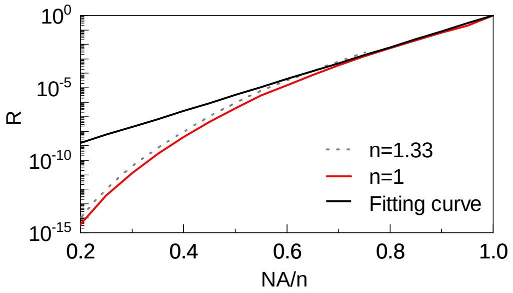

Fig. S10: The ratio R of the minimum and maximum intensities P_{signal} as a function of NA/n. Red solid curve: n=1; Gray dashed curve: n=1.33; Black solid curve: Fitting curve: $R \approx$110 (NA/n − 1) dB.

Supplementary Note 3: Scattering model

The nanoscale surface roughness, boundary deformations or external particles could scatter part of emitted and intracavity light to directions out of the disk plane by elastic scattering. The Rayleigh-scattered intensity $P_{\text{sc}}(\alpha)$ should have the maximum when $\alpha = 0°$ and the minimum at 90° because the electric field of the TE mode is polarized primarily in the plane of the disk (Fig. S3a). Integrating the random dipole-induced scattered light of the TE mode over the entire surface of a microdisk, the scattering pattern could be heuristically described as the Rayleigh scattering profile for unpolarized light:

$$|E_{\text{sc}}(\theta,\phi)|^2 \propto 1 + \cos^2\theta, \tag{S3}$$

While a conventional LP predominantly emits in the radial direction, scattering predominantly radiates orthogonally to this plane about the $\theta = 0°$ axis, which we define as the +z axis. Notice that we can rewrite the angle dependence of the scattering radiation in terms of the spherical unit vector $\vec{r} = \sin\theta\,\cos\phi\;\vec{x} + \sin\theta\,\sin\phi\;\vec{y} + \cos\theta\;\vec{z}$.

$$|E_{\text{sc}}(\vec{r})|^2 \propto 1 + (\vec{r}\cdot\vec{z})^2, \tag{S4}$$

To account for rotation of the microdisk LP, we use a standard rotation matrix $\mathbb{R}$ to rotate $\vec{r}$ about the x-axis by some angle α to yield a new vector

$$\vec{r}_\alpha = \mathbb{R}(\alpha)\vec{r} = \sin\theta\,\cos\phi\;\vec{x} + (\cos\alpha\,\sin\theta\,\sin\phi - \sin\alpha\,\cos\theta)\;\vec{y} + (\sin\alpha\,\sin\theta\,\sin\phi + \cos\alpha\,\cos\theta)\;\vec{z} \tag{S5}$$

Therefore, we expect the number of scattered photons collected by our optical setup for a disk tilted from horizontal by an angle α to be given by

$$P_{\text{sc}}(\alpha) \propto \int_0^{\theta_0}\int_0^{2\pi} |E_{\text{sc}}(\theta,\phi)|^2 \sin\theta\,\mathrm{d}\theta\,\mathrm{d}\phi, \tag{S6}$$

where θ_0 defines the collection angle of the objective. Evaluating this integral gives

$$\begin{aligned} P_{\text{sc}}(\alpha) &\propto \frac{\pi}{24}(\cos 2\alpha[3\cos\theta_0 - 3\cos 3\theta_0] - 63\cos\theta_0 - \cos 3\theta_0 + 64) \\ &\approx \frac{\pi\theta_0^2}{2}(3 + \cos 2\alpha) \\ &= \pi\theta_0^2(1 + \cos^2\alpha) \end{aligned} \tag{S7}$$

where going from the first to second line we have used the paraxial approximation $\cos x = 1 - x^2/2$. This suggests that a sinusoidally quadratic emission pattern retains the general form of its angle dependence when its emission is collected by a paraxial optical system, i.e.

$$P_{\text{sc}}(\alpha) \propto 1 + \cos^2\alpha \tag{S8}$$

Combining the in-plane emission from the microdisk and the out-of-plane scattering from the perturbations, the total pattern of OLP can be expressed as

$$P_{\rm tot}(\alpha) = (1-s)\cdot P_0(\alpha) + s\cdot P_{\rm sc}(\alpha), \tag{S9}$$

where $P_0(\alpha)$ is the intrinsic emission of the lasing mode in the perturbed LP, $P_{\rm sc}(\alpha)$ denotes the intensity pattern arising from scattering objects, and s represents the fraction of light scattered. If the perturbation to the lasing mode is small, $P_0(\alpha)$ would be close to P_{signal}. The Rayleigh-scattered intensity $P_{\rm sc}(\alpha)$ should have the maximum at $\alpha = 0°$ and the minimum at 90°. The original and scattered profiles are thus complementary and could constitute omnidirectional emission for sufficiently large s. With appropriate normalization factors, Eq. (S9) may be written as, for NA < 0.7:

$$P_{\rm tot}(\alpha) \propto (1-s)\cdot {\rm c}_0\ |{\rm sin}\alpha|^q + s\cdot {\rm c}_1(1+\cos^2\alpha), \tag{S10}$$

where ${\rm c}_0 = \int^{4\pi} \sin^{18}\theta\, {\rm d}\Omega / \int^{4\pi} \sin^q\theta\, {\rm d}\Omega \approx 0.75$ for NA $= 0.45$ ($q = 10$) and ${\rm c}_1 = \int^{4\pi} \sin^{18}\theta\, {\rm d}\Omega / \int^{4\pi} (1+\cos^2\theta)\, {\rm d}\Omega \approx 0.21$, ${\rm c}_1/{\rm c}_0 = \int^{4\pi} \sin^q\theta\, {\rm d}\Omega \,/\, \int^{4\pi} (1+\cos^2\theta)\, {\rm d}\Omega \approx 0.28$. Putting these values into Eq. (S10), we find the min-max ratio to be:

$$R \approx \frac{0.56s}{1-0.72s}. \tag{S11}$$

The criteria of $R > 0.01$ and $R > 0.1$ are satisfied when $s > 0.018$ and $s > 0.16$, respectively.

Supplementary Note 4: scLPs obtained by chemical functionalization

We have developed silica coating on LPs by a modified Stöber method. Therefore, we can also adsorb scatterers onto LP templates by a chemical bonding method, which has widely been used to conjugate various nanoparticles and biomolecules onto silica surface with chemical modifications. Firstly, a numerical simulation was performed to illustrate the feasibility of this approach. As an example, 47 nanospheres with a diameter of 200 nm are randomly distributed on the surface of a LP with a 50-nm-thick silica gap, as shown in the device models (Fig. S6a). The simulated $P_{\rm tot}(\alpha)$ (Fig. S6b) of the TE mode shows a distinct emission in the vertical direction. The vertical output becomes larger with increasing refractive index of the nanoparticles, while the scatterers do not dramatically degrade the Q-factor of the cavity (Fig. S6c).

In the experiment, we used commercially-available TiO_2 nanospheres functionalized with carboxyl groups as scatterers on silica coated LPs. The scLP production process is described in Fig. S6d, and involves conjugating a great number of carboxyl TiO_2 particles onto the amino-functionalized silica surface around LPs through amide bond formation. As a result, hundreds of TiO_2 particles with a diameter of about 200 nm were coated on the surface of LPs (Fig. S6f). Measurements of scLPs suspended in the hydrogel show no distinct improvement in the dynamic range R, with both scLPs and control LPs failing to meet the omnidirectionality criterion $R > 0.01$ (Figs. S6g-h). This is attributed to the low refractive index (< 1.8) of amorphous TiO_2 nanoparticles and the large silica gap distance ($\sim$ 50 nm) between TiO_2 particles and semiconductor disk surface. Although the TiO_2 nanoparticles have no distinct scattering effect in hydrogel, this developed chemical bonding method could be suitable for high-refractive-index nanoparticles as scatterers on LPs.

Methods of scLPs obtained by chemical functionalization.

Silica coating: Silica coating of the microdisks was performed by a modified Stöber process. A typical silica coating with a thickness of 50 nm is as follows [3]: Microdisks (about 10^5 LPs/ml) were suspended in 670 µl of ethanol:H_2O solution (80 v/v% ethanol). Next, 60 µl of 40 mM tetraethyl orthosilicate (TEOS) in ethanol, and 45 µl of ammonium hydroxide solution (28 v/v% NH4OH) were added, and the microdisk solution was shaken vigorously at 1,400 rpm. for 1 h at room temperature. To harden the silica shell and improve chemical stability, the temperature was increased to 70°C and the solution was mixed for an additional 2-12 hours. Then, the microdisks were filtered out by a transwell centrifuge filter with a pore size of 1 µm. To remove the small silica nanoparticles, the microdisks in the transwell centrifuge filter were sonicated for 5-10 mins in DI water and thoroughly centrifuge-filtered 3-4 times. Amino functionalization: The silica-coated microdisk was suspended in 950 µl of ethanol solution. Then, 40 µl of NH_4OH and 10 µl of (3-aminopropyl)-triethoxysilane (APTES) were added, and the microdisk solution was shaken vigorously at 1,400 r.p.m. overnight at room temperature. The suspension of microdisks was then transferred to a 1 µm-pore centrifuge filter, and filtered thoroughly by at least three repeated cycles of centrifugation and resuspension (via ultrasonication) using ethanol and DI water.

TiO_2 nanoparticle coating: Carboxylated titania nanoparticles with a diameter of 200 nm (10^{10} nanoparticles/ml, Microspheres-Nanospheres company) in water was firstly filtered using a centrifuge filter with a pore size of 1 µm to remove the aggregated nanoparticles. Carboxylated TiO_2 particles and amino-functionalized microdisks were separately dispersed in buffer solution (300 µl). Buffer solution refers to MES aqueous solution (10 mM, pH=5). A microdisk solution was then added to the TiO_2 particles solution with ultrasonic treatment and placed on a shaker for 5 minutes. A buffer solution (200 µl) containing N-(3-dimethylaminopropyl)-N'-ethylcarbodiimide Hydrochloride (EDC, 25 mg) and N-hydroxysuccinimide (NHS, 25 mg) was added and placed on a shaker for another 3 hours. Finally, the suspension of nanoparticle-coated microdisks was then transferred to a 1 µm-pore centrifuge filter, and filtered thoroughly by at least five repeated cycles of centrifugation and resuspension (via ultrasonication) using DI water.

Supplementary Note 5: Effect of deep tissue scattering on angle-dependent collection efficiency.
In applications in which microdisk LPs are imaged deep within biological tissues, intrinsic scattering of light from the tissue itself may effect the angle-dependent collection efficiency of the LP. To investigate this phenomenon, an open-source Monte-Carlo simulation platform was used[4]. By defining a directional emitter of the theoretical form $|E_{\mathrm{far}}(\theta)|^2 = \sin^{18}(\theta)$ (Supplementary Note 1) using custom C code, we were able to simulate disk emission for a variety of angles between $\theta = 0$ and $\theta = 90°$. The tissue scattering properties were chosen to be consistent with typical values for brain tissue at 1270 nm with a scattering coefficient $\mu_s = 72$ cm^{-1} and anisotropy $g = 0.9$[5]. The absorption coefficient $\mu_a = 1.1$ cm^{-1} was set to be that of water[6].

Figure S11a shows 100 sample photon paths for a microdisk located at $(x, y, z) = (0, 0, 0.3$ mm$)$ viewed along the y-axis with angle $\alpha = 0$ (i.e. a flat disk). The surface of the tissue is located at the $z = 0$ plane. Figure S11b shows the analogous case but for a vertically oriented disk $\alpha = 90°$. To investigate the role of angle dependence on collection efficiency in scattering tissue, a 3 cm x 3 cm x 3 cm simulation volume was used. Collection was simulated by saving the paths of between 0.6×10^6 and 10^6 photons for a variety of starting disk tilt angles and starting depths. To determine whether photons leaving the tissue were successfully collected, a custom Matlab script simulated lossless propagation between the objective lens and the spectrometer's linescan detector with a pixel size of 10 μm (along the array) by 210 μm (height). Perfect NA matching between the 0.45 NA objective and 0.13 NA collection aperture of our spectrometer was assumed. The internal spectrometer magnification was set to unity and the slit size at 20 μm and pixel height at 210 μm.

The results are shown in Figure S11c. Even at depths up to 500 μm, the collection efficiency of a vertically oriented disk far exceeds that of a flat disk. Fitting curves for each initial depth is shown, resulting in estimates of $s = 0.004$, $s = 0.006$ and $s = 0.01$ corresponding to $R = -26.5$ dB, $R = -24.7$ dB, and $R = -22.5$ dB for depths of 100 μm, 300 μm and 500 μm respectively. At these depths, collection of light from the microdisk is still highly angle-dependent and does not fulfill our goal of $R < 0.01$.

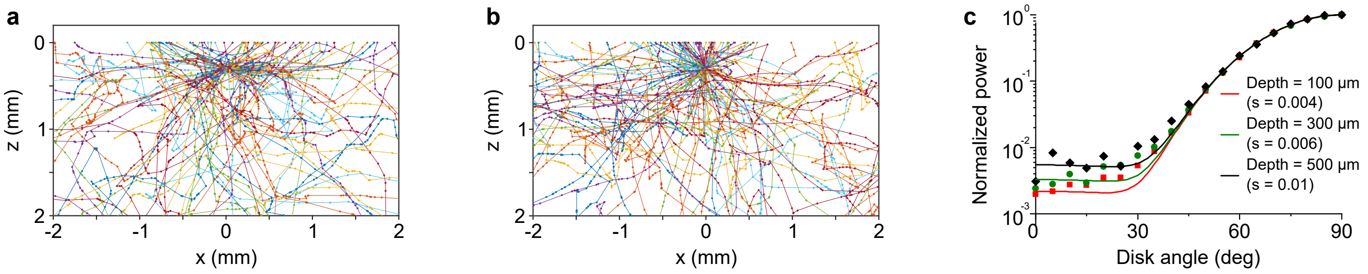

Fig. S11: The effect of tissue scattering on angle dependent collection. Sample photon paths from a Monte Carlo simulation for **a** a flat disk situated at depth $z = 0.3$ mm, and **b** a vertical disk situated at a depth $z = 0.3$ mm. **c**, The normalized power collected at different angles for depths 0.1, 0.3 and 0.5 mm with fitting curves (See Supplementary Note 3).

Supplementary Video

Supplementary video: A microdisk laser particle internalized by a cell. This video shows that the orientation of the loaded LP varies over time as the cell moves, resulting in random disk orientations at any given moment.

[1] Lee, T. D. *et al.* Far-field emission narrowing effect of microdisk lasers. *Appl. Phys. Lett.* **72**, 2223–2225 (1998).

[2] Lee, T.-D. *et al.* Measurement of far-field emission of semiconductor micro-disk lasers. In *Conference on Lasers and Electro-Optics*, CWF31 (Optical Society of America, 1997).

[3] Martino, N. *et al.* Wavelength-encoded laser particles for massively multiplexed cell tagging. *Nat. Photon.* **13**, 720 (2019).

[4] Marti, D., Aasbjerg, R. N., Andersen, P. E. & Hansen, A. K. MCmatlab: an open-source, user-friendly, MATLAB-integrated three-dimensional Monte Carlo light transport solver with heat diffusion and tissue damage. *Journal of Biomedical Optics* **23**, 1 (2018).

[5] Jacques, S. L. Optical properties of biological tissues: a review. *Phys. Med. Biol* **58**, 37–61 (2013).

[6] Kou, L., Labrie, D. & Chylek, P. Refractive indices of water and ice in the 065- to 25-μm spectral range. *Applied Optics* **32**, 3531 (1993).
